# Supplementary figures and images for: Is there a causal relationship between resistin levels and bone mineral density, fracture occurrence? A mendelian randomization study
Source: PLoS One. 2024 Aug 27;19(8):e0305214. doi: 10.1371/journal.pone.0305214 (PMC11349205; doi:10.1371/journal.pone.0305214)

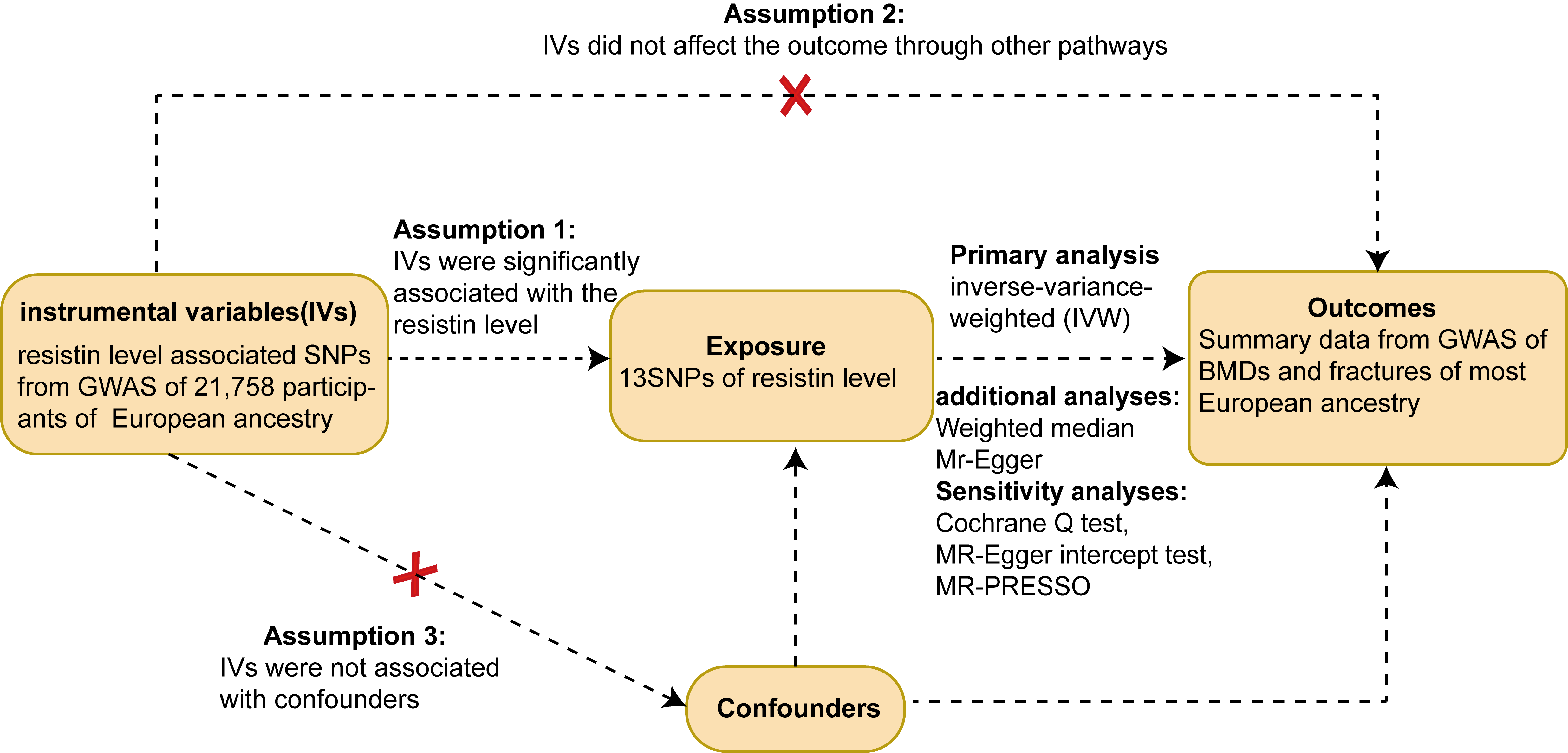

Supplement: S1 Fig — (TIF) [file pone.0305214.s001.tif]

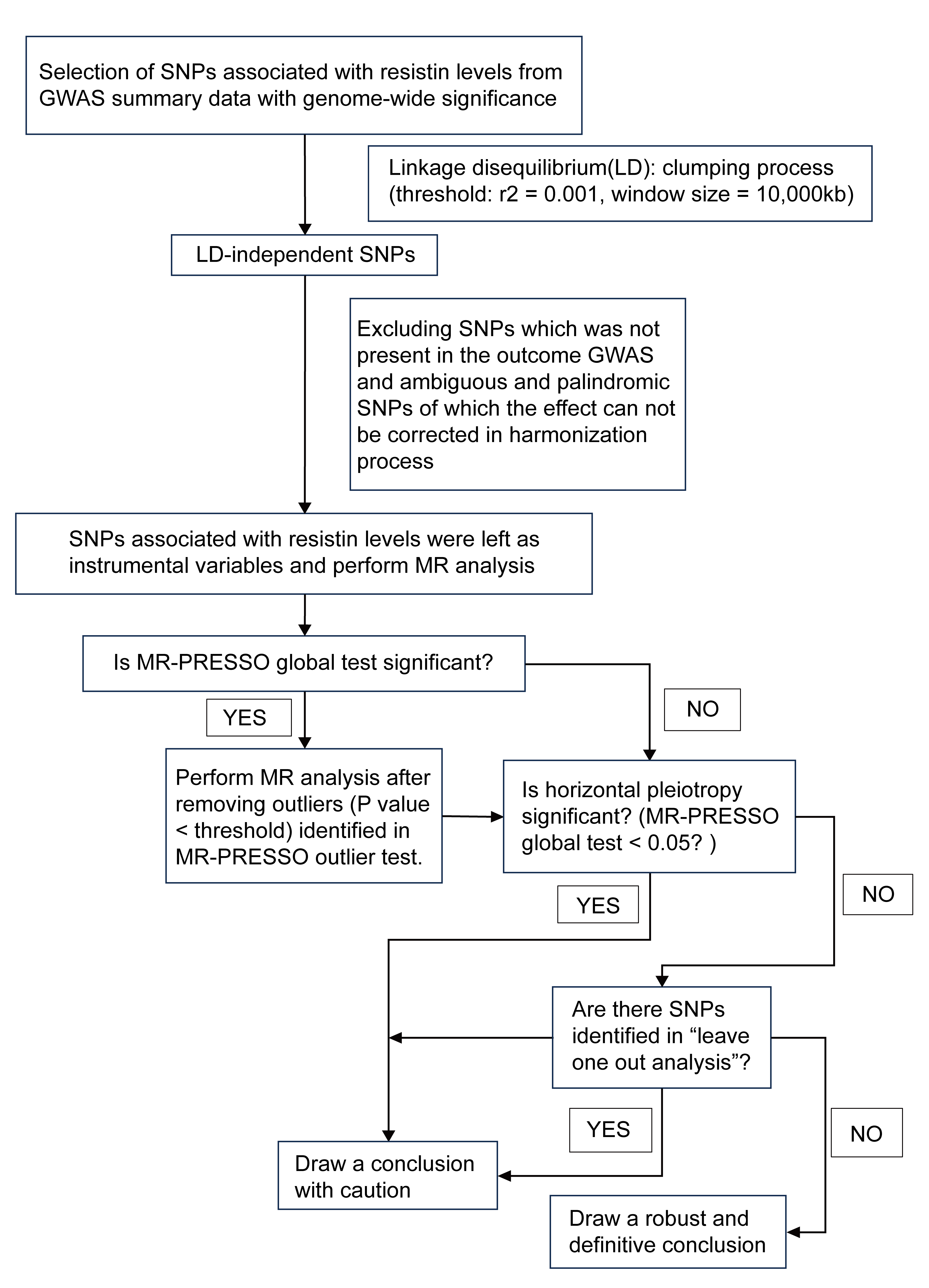

Supplement: S2 Fig — (TIF) [file pone.0305214.s002.tif]

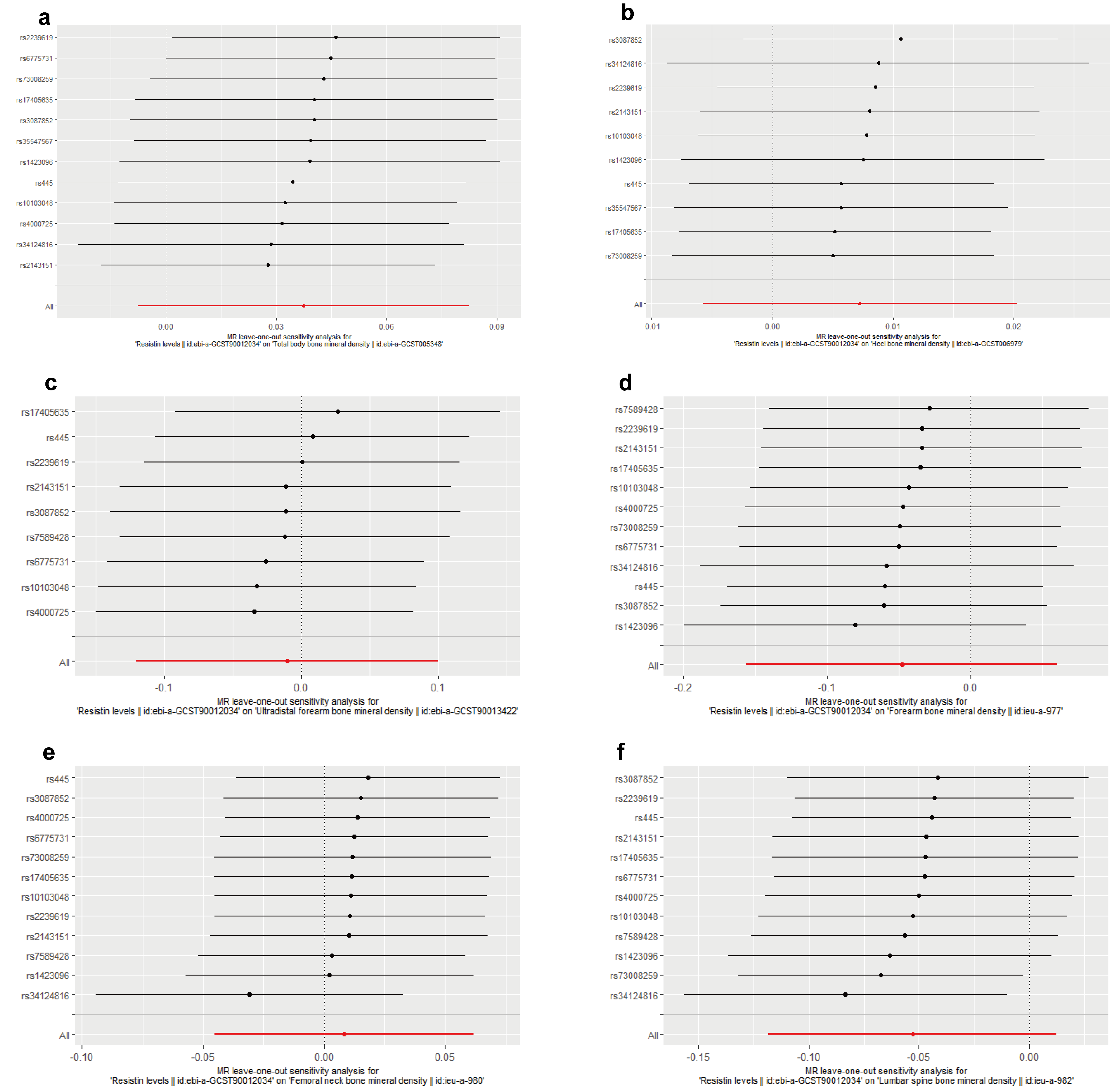

Supplement: S3 Fig — (a) TB-BMD; (b) HE-BMD; (c) UF-BMD; (d) FA-DMD; (e) FN-BMD; (f) LS-BMD. (TIF) [file pone.0305214.s003.tif]

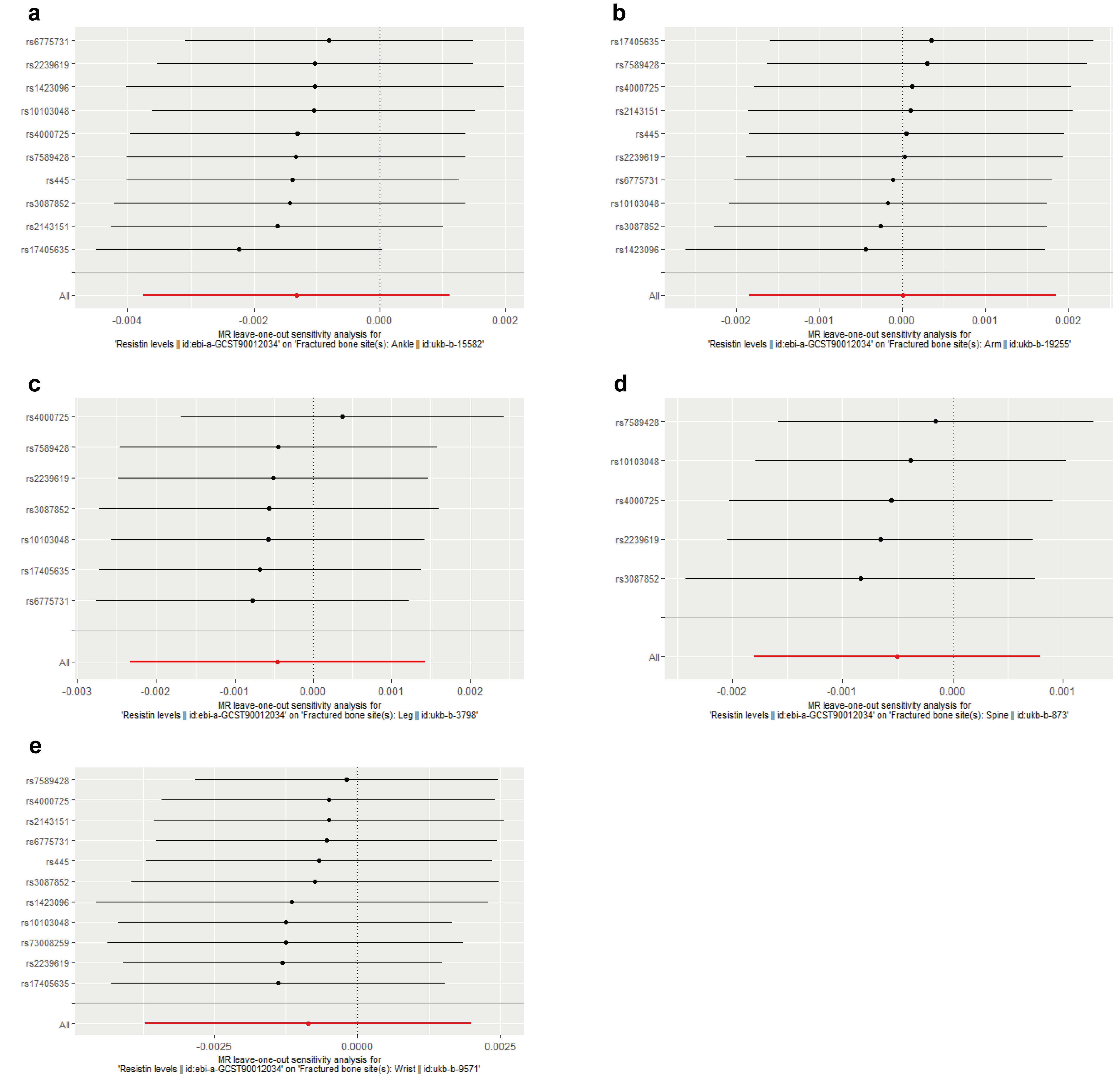

Supplement: S4 Fig — (a) ankle fracture; (b) arm fracture; (c) leg fracture; (d) spine fracture; (e) wrist fracture. (TIF) [file pone.0305214.s004.tif]

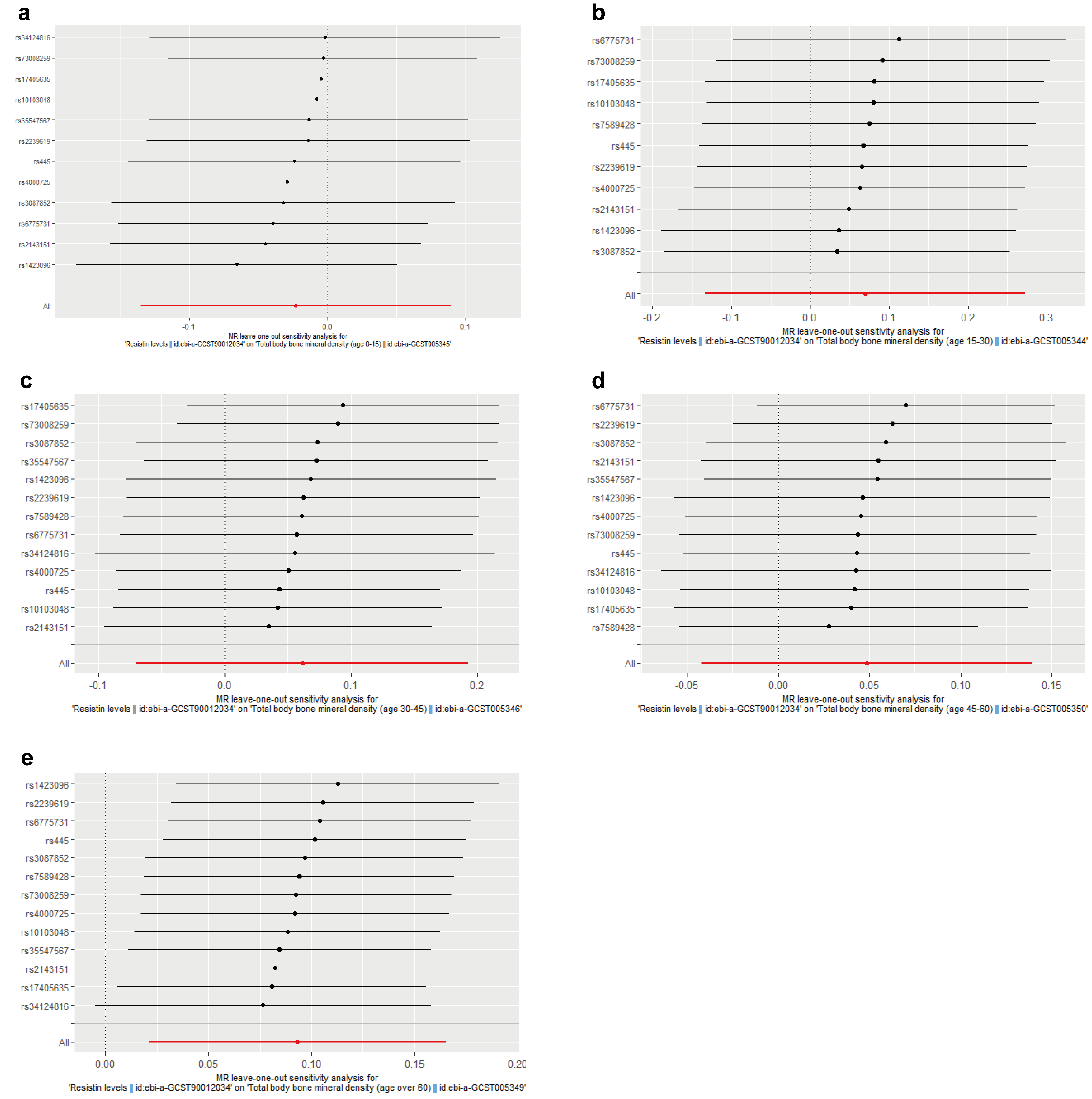

Supplement: S5 Fig — (a) age 0–15; (b) age 15–30; (c) age 30–45; (d) age 45–60; (e) age > 60. (TIF) [file pone.0305214.s005.tif]

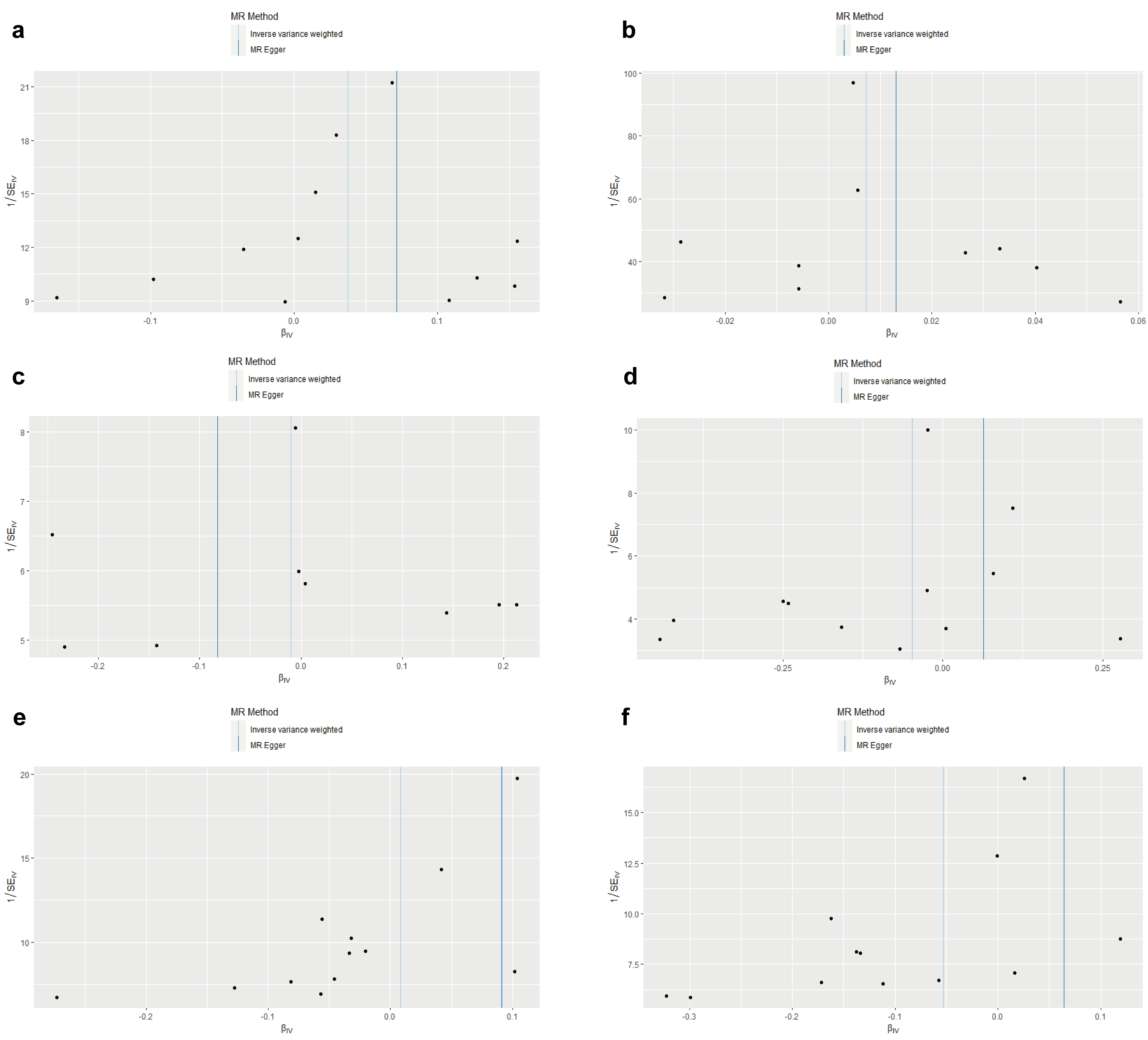

Supplement: S6 Fig — (a) TB-BMD; (b) HE-BMD; (c) UF-BMD; (d) FA-DMD; (e) FN-BMD; (f) LS-BMD. (TIF) [file pone.0305214.s006.tif]

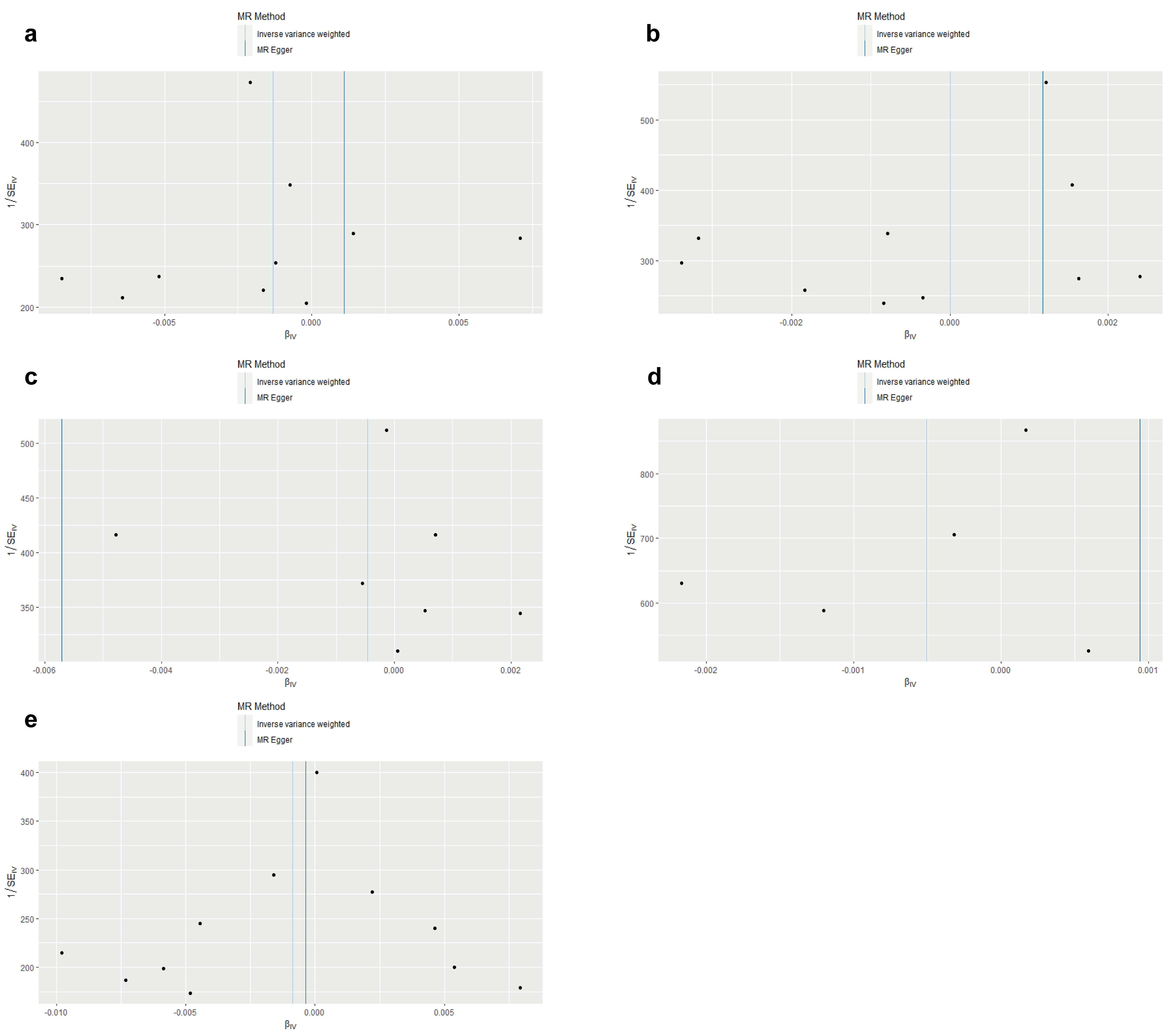

Supplement: S7 Fig — (a) ankle fracture; (b) arm fracture; (c) leg fracture; (d) spine fracture; (e) wrist fracture. (TIF) [file pone.0305214.s007.tif]

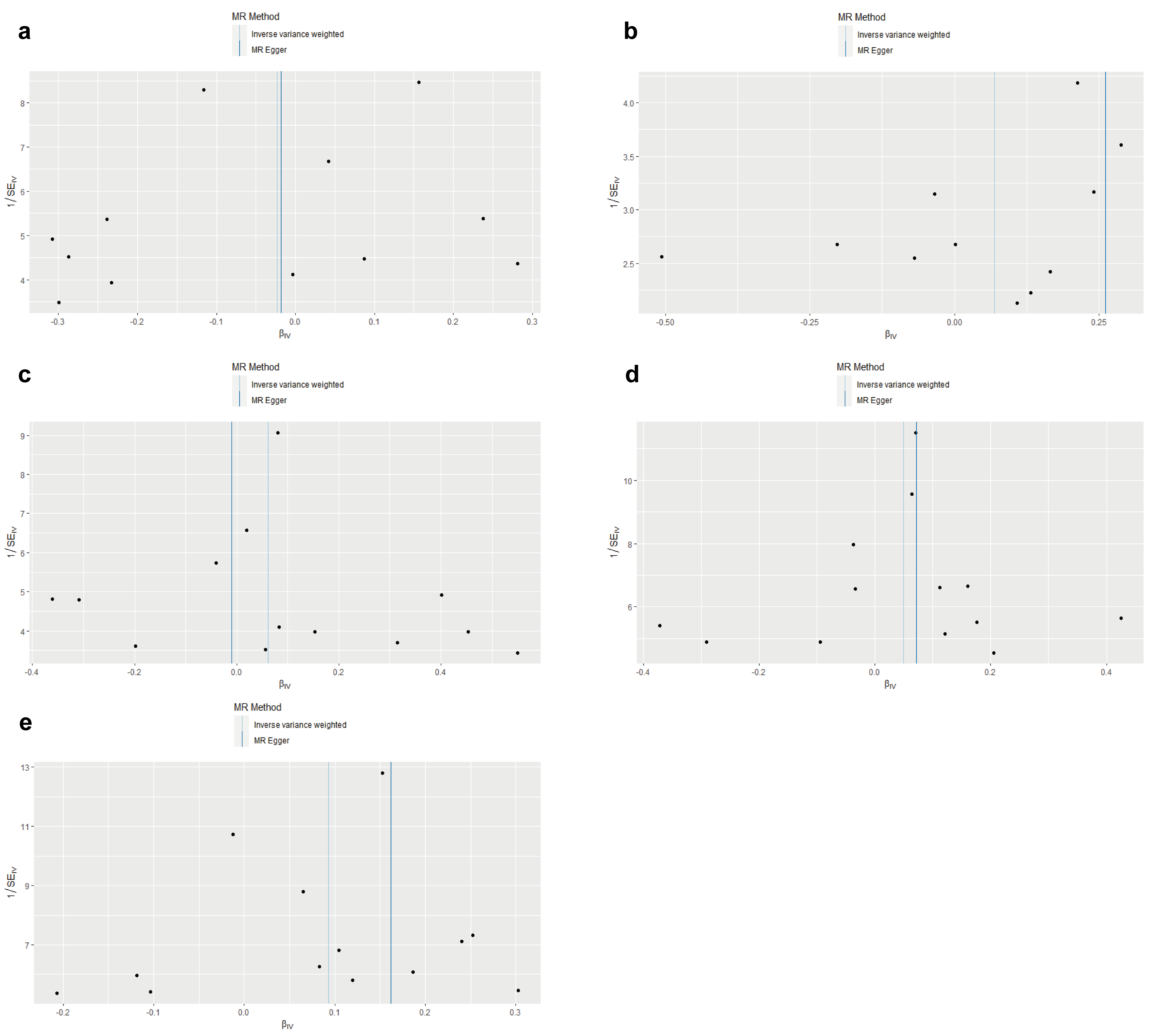

Supplement: S8 Fig — (a) age 0–15; (b) age 15–30; (c) age 30–45; (d) age 45–60; (e) age > 60. (TIF) [file pone.0305214.s008.tif]
