# Supplementary material for: Is there a causal relationship between resistin levels and bone mineral density, fracture occurrence? A mendelian randomization study
Source: PLoS One. 2024 Aug 27;19(8):e0305214. doi: 10.1371/journal.pone.0305214 (PMC11349205; doi:10.1371/journal.pone.0305214)
Supplement: S1 Table — (DOCX) [file pone.0305214.s009.docx]

**S1** **Table. Characteristics of GWAS data of exposure and outcome.**

| **phenotype** | **Sample size** | **Data source** | **ancestry** | **Phenotype description** | **GWAS ID** | **PMID** |
| --- | --- | --- | --- | --- | --- | --- |
| Resistin | 21,758 | SCALLOP consortium | European | Resistin levels | ebi-a-GCST90012034 | 33067605 |
| TB-BMD | 56,284 | GEFOS consortium | European | Total body bone mineral density | ebi-a-GCST005348 | 29304378 |
| HE-BMD | 426,824 | UK-Biobank | European | Heel bone mineral density | ebi-a-GCST006979 | 30598549 |
| UF-BMD | 21,907 | NA | European | Ultradistal forearm bone mineral density | ebi-a-GCST90013422 | 33097703 |
| FA-BMD | 8,143 | GEFOS consortium | European | Forearm bone mineral density | ieu-a-977 | 26367794 |
| FN-BMD | 32,735 | GEFOS consortium | European | Femoral neck bone mineral density | ieu-a-980 | 26367794 |
| LS-BMD | 28,498 | GEFOS consortium | European | Lumbar spine bone mineral density | ieu-a-982 | 26367794 |
| ankle | 460,340 | MRC-IEU | European | Fractured bone site(s): Ankle | ukb-b-15582 | NA |
| arm | 460,340 | MRC-IEU | European | Fractured bone site(s): Arm | ukb-b-19255 | NA |
| leg | 460,340 | MRC-IEU | European | Fractured bone site(s): Leg | ukb-b-3798 | NA |
| spine | 460,340 | MRC-IEU | European | Fractured bone site(s): Spine | ukb-b-873 | NA |
| wrist | 460,340 | MRC-IEU | European | Fractured bone site(s): Wrist | ukb-b-9571 | NA |
| age 0–15 | 11,807 | GEFOS consortium | 86% European ancestry | Total body bone mineral density (age 0–15) | ebi-a-GCST005345 | 29304378 |
| age 15–30 | 4,180 | GEFOS consortium | 86% European ancestry | Total body bone mineral density (age 15–30) | ebi-a-GCST005344 | 29304378 |
| age 30–45 | 10,062 | GEFOS consortium | 86% European ancestry | Total body bone mineral density (age 30–45) | ebi-a-GCST005346 | 29304378 |
| age 45–60 | 18,805 | GEFOS consortium | 86% European ancestry | Total body bone mineral density (age 45–60) | ebi-a-GCST005350 | 29304378 |
| age over 60 | 22,504 | GEFOS consortium | 86% European ancestry | Total body bone mineral density (age over 60) | ebi-a-GCST005349 | 29304378 |

**Abbreviation:** SCALLOP, Systematic and Combined AnaLysis of Olink Proteins; GEFOS, GEnetic Factors for Osteoporosis; MRC-IEU, Medical Research Council Integrative Epidemiology Unit; GWAS, genome wide association studies.
