# Supplementary material for: Is there a causal relationship between resistin levels and bone mineral density, fracture occurrence? A mendelian randomization study
Source: PLoS One. 2024 Aug 27;19(8):e0305214. doi: 10.1371/journal.pone.0305214 (PMC11349205; doi:10.1371/journal.pone.0305214)
Supplement: S2 Table — (DOCX) [file pone.0305214.s010.docx]

**S2 Table. Detailed information of LD-independent SNPs (after clumping process) for exposure (resistin levels).**

| **SNP** | **Effect allele** | **Other allele** | **beta** | **se** | **eaf** | **Sample size** | **p-value** |
| --- | --- | --- | --- | --- | --- | --- | --- |
| *rs4000725* | C | A | -0.0905 | 0.0165 | 0.8548 | 21758 | 3.86E-08 |
| *rs17405635* | A | G | 0.0799 | 0.0107 | 0.2625 | 21758 | 6.61E-14 |
| *rs7589428* | G | A | -0.0626 | 0.0095 | 0.491 | 21758 | 4.64E-11 |
| *rs6775731* | C | T | 0.0632 | 0.0107 | 0.6978 | 21758 | 3.17E-09 |
| *rs73008259* | A | G | 0.1926 | 0.0204 | 0.0527 | 21758 | 3.68E-21 |
| *rs2143151* | G | T | 0.121 | 0.0162 | 0.9131 | 21758 | 7.27E-14 |
| *rs2239619* | A | C | -0.0532 | 0.0097 | 0.6221 | 21758 | 4.06E-08 |
| *rs445* | T | C | -0.085 | 0.0154 | 0.1008 | 21758 | 3.30E-08 |
| *rs10103048* | C | A | -0.0596 | 0.0096 | 0.5849 | 21758 | 5.25E-10 |
| *rs3087852* | A | G | -0.0861 | 0.0094 | 0.4597 | 21758 | 6.60E-20 |
| *rs34124816* | C | A | -0.4782 | 0.0345 | 0.0409 | 21758 | 8.43E-44 |
| *rs1423096* | C | T | -0.1886 | 0.0167 | 0.9072 | 21758 | 2.05E-29 |
| *rs35547567* | T | C | 0.3352 | 0.0585 | 0.0154 | 21758 | 1.00E-08 |

**Abbreviation:** LD, linkage disequilibrium; SNP, single nucleotide polymorphis。
