# Supplementary material for: Is there a causal relationship between resistin levels and bone mineral density, fracture occurrence? A mendelian randomization study
Source: PLoS One. 2024 Aug 27;19(8):e0305214. doi: 10.1371/journal.pone.0305214 (PMC11349205; doi:10.1371/journal.pone.0305214)
Supplement: S3 Table — (DOCX) [file pone.0305214.s011.docx]

**S3 Table****. *F*-statistics of instrumental variables (IVs).**

| exposure | Sample size | nIV | R^2^ | *F*-statistics |
| --- | --- | --- | --- | --- |
| Resistin levels  ID: ebi-a-GCST90012034 | 21758 | 13 | 0.03864222 | 67.231543 |
