# Supplementary material for: Is there a causal relationship between resistin levels and bone mineral density, fracture occurrence? A mendelian randomization study
Source: PLoS One. 2024 Aug 27;19(8):e0305214. doi: 10.1371/journal.pone.0305214 (PMC11349205; doi:10.1371/journal.pone.0305214)
Supplement: S4 Table — (DOCX) [file pone.0305214.s012.docx]

**S4 Table. Sensitivity analysis outcome of all analyses (after removing outliers).**

| Outcome |  | Q Pvalue | |  | Egger intercept p | |  | PRESSO  Global p | Steiger  test |
| --- | --- | --- | --- | --- | --- | --- | --- | --- | --- |
|  |  | Q | P-value |  | intercept | P-value |  |  |  |
| TB-BMD |  | 11.72 | 0.38 |  | -0.00411 | 0.38 |  | 0.451 | TRUE |
| HE-BMD |  | 74.55 | 0.52 |  | -0.00095 | 0.52 |  | 0.436 | TRUE |
| UF-BMD |  | 7.48 | 0.79 |  | 0.00545 | 0.79 |  | 0.51 | TRUE |
| FA-BMD |  | 8.89 | 0.2 |  | -0.01429 | 0.2 |  | 0.641 | TRUE |
| FN-BMD |  | 10.73 | 0.07 |  | -0.01026 | 0.07 |  | 0.395 | TRUE |
| LS-BMD |  | 12.14 | 0.035 |  | -0.01442 | 0.035 |  | 0.368 | TRUE |
| ankle fracture |  | 11.38 | 0.47 |  | -2.21E-04 | 0.47 |  | 0.302 | TRUE |
| arm fracture |  | 3.95 | 0.64 |  | -1.07E-04 | 0.64 |  | 0.917 | TRUE |
| leg fracture |  | 4.46 | 0.35 |  | 3.78E-04 | 0.35 |  | 0.612 | TRUE |
| spine fracture |  | 1.95 | 0.68 |  | -1.04E-04 | 0.68 |  | 0.758 | TRUE |
| wrist fracture |  | 14.06 | 0.83 |  | -4.70E-05 | 0.83 |  | 0.213 | TRUE |
| TB-BMD (age 0–15) |  | 13.4 | 0.48 |  | -0.01742 | 0.48 |  | 0.93 | TRUE |
| TB-BMD (age 15–30) |  | 4.32 | 0.96 |  | -0.00055 | 0.96 |  | 0.268 | TRUE |
| TB-BMD (age 30–45) |  | 17.68 | 0.51 |  | 0.00865 | 0.51 |  | 0.15 | TRUE |
| TB-BMD (age 45–60) |  | 11.37 | 0.27 |  | -0.00798 | 0.27 |  | 0.516 | TRUE |
| TB-BMD (age > 60) |  | 15.61 | 0.78 |  | -0.00263 | 0.78 |  | 0.276 | TRUE |

**Abbreviation:** TB-BMD, total body bone mineral density; HE-BMD, Heel bone mineral density; UF-BMD, Ultradistal forearm bone mineral density; FA-BMD, Forearm bone mineral density; FN-BMD, Femoral neck bone mineral density; LS-BMD, Lumbar spine bone mineral density.
