# Supplementary material for: Is there a causal relationship between resistin levels and bone mineral density, fracture occurrence? A mendelian randomization study
Source: PLoS One. 2024 Aug 27;19(8):e0305214. doi: 10.1371/journal.pone.0305214 (PMC11349205; doi:10.1371/journal.pone.0305214)
Supplement: S5 Table — (DOCX) [file pone.0305214.s013.docx]

**S5 Table. Characterization of GWAS statistics for confounding factors.**

| **phenotype** | **Sample size** | **Data source** | **ancestry** | **GWAS ID** | **PMID** |
| --- | --- | --- | --- | --- | --- |
| Physical activity | 89,683 | UK-Biobank | European | ebi-a-GCST90093322 | 34753499 |
| Smoking status: Previous | 336,024 | Neale Lab | European | ukb-a-224 | NA |
| alcohol consumption | 83,626 | Within family GWAS consortium | European | ieu-b-4834 | NA |
| Serum 25-Hydroxyvitamin D levels | 443,734 | UK-Biobank | European | ebi-a-GCST010144 | 32059762 |
